# PM2b, a CC-NBS-LRR protein, interacts with TaWRKY76-D to regulate powdery mildew resistance in common wheat

**DOI:** 10.3389/fpls.2022.973065

**Published:** 2022-10-26

**Authors:** Yuli Jin, Hong Liu, Tiantian Gu, Lixian Xing, Guohao Han, Pengtao Ma, Xiuquan Li, Yilin Zhou, Jieru Fan, Lihui Li, Diaoguo An

**Affiliations:** ^1^ Center for Agricultural Resources Research, Institute of Genetics and Developmental Biology, Chinese Academy of Sciences, Shijiazhuang, Hebei, China; ^2^ The National Key Facility for Crop Gene Resources and Genetic Improvement, Institute of Crop Science, Chinese Academy of Agricultural Sciences, Beijing, China; ^3^ The State Key Laboratory for Biology of Plant Disease and Insect Pests, Institute of Plant Protection, Chinese Academy of Agricultural Sciences, Beijing, China; ^4^ The Innovative Academy for Seed Design, Chinese Academy of Sciences, Beijing, China

**Keywords:** *Pm2b*, *TaWRKY76-D*, wheat, MAS, powdery mildew

## Abstract

Powdery mildew caused by *Blumeria graminis* f. sp. *tritici* (*Bgt*) is a destructive disease of wheat throughout the world. Host resistance is considered the most sustainable way to control this disease. Powdery mildew resistance gene *Pm2b* was mapped to the same genetic interval with *Pm2a* and *PmCH1357* cloned previously, but showed different resistance spectra from them, indicating that they might be caused by different resistance genes or alleles. In this study, *Pm2b* was delimited to a 1.64 Mb physical interval using a large segregating population containing 4,354 F_2:3_ families of resistant parent KM2939 and susceptible cultivar Shimai 15. In this interval, *TraesCS5D03G0111700* encoding the coiled-coil nucleotide-binding site leucine-rich repeat protein (CC-NBS-LRR) was determined as the candidate gene of *Pm2b*. Silencing by barley stripe mosaic virus-induced gene silencing (BSMV-VIGS) technology and two independent mutants analysis in KM2939 confirmed the candidate gene *TraesCS5D03G0111700* was *Pm2b*. The sequence of *Pm2b* was consistent with *Pm2a/PmCH1357.* Subcellular localization showed *Pm2b* was located on the cell nucleus and plasma membrane. *Pm2b* had the highest expression level in leaves and was rapidly up-regulated after inoculating with *Bgt* isolate E09. The yeast two-hybrid (Y2H) and luciferase complementation imaging assays (LCI) showed that PM2b could self-associate through the NB domain. Notably, we identified PM2b interacting with the transcription factor TaWRKY76-D, which depended on the NB domain of PM2b and WRKY domain of TaWRKY76-D. TaWRKY76-D negatively regulated the resistance to powdery mildew in wheat. The specific KASP marker *K529* could take the advantage of high-throughput and high-efficiency for detecting *Pm2b* and be useful in molecular marker assisted-selection breeding. In conclusion, cloning and disease resistance mechanism analysis of *Pm2b* provided an example to emphasize a need of the molecular isolation of resistance genes, which has implications in marker assisted wheat breeding.

## Introduction

Wheat (*Triticum aestivum* L., 2n=6x=42, AABBDD) is one of the most important food crops in the world and provides 20% of the total daily calories and protein consumed by humankind (Reynolds et al., 2012). Diseases and pests lead to a serious threat to the global production of wheat grain and food supply (Savary et al., 2019). Powdery mildew caused by *Blumeria graminis* f. sp. *tritici* (*Bgt*) is a destructive disease of wheat worldwide (Gao et al., 2018; Singh et al., 2016). Chemical prevention is usually used to control this disease, but will increase the cost, accelerate the variation of *Bgt* isolates and endanger the environment (Bennett, 1984; Bliffeld et al., 1999). Host resistance is the most economical, efficient, and environmental-friendly method to prevent powdery mildew (Koller et al., 2018; Sun et al., 2018). To date, 68 powdery mildew resistance genes (*Pm*) at 63 loci (*Pm1*-*Pm68*) (*Pm8* = *Pm17*, *Pm18* = *Pm1c*, *Pm22* = *Pm1e*, *Pm23* = *Pm4c*, and *Pm31* = *Pm21*) have been identified from diversified genotypes (McIntosh et al., 2019; He et al., 2020).

Among the reported *Pm* loci, an interesting multi-allelic phenomenon is that seven *Pm* loci (*Pm1a*-*Pm1e*, *Pm2a*-*Pm2c*, *Pm3a-3j*, *Pm4a-4d*, *Pm5a-5e*, *Pm24a-24b* and *Pm60-60b*) have more than one resistance allele (Qie et al., 2019; Zou et al., 2022). Studies of multi-allelic race-specific *R* genes reveal that they evolve to recognize specific avirulence (*Avr*) alleles from the pathogen under strong diversifying selection (Rose et al., 2004; Bhullar et al., 2009; Kanzaki et al., 2012). For instance, 17 functional alleles confer race-specific resistance to powdery mildew at *Pm3* locus (Bhullar et al., 2009; Brunner et al., 2010). PM3 belongs to the sub-group of nucleotide binding site and leucine rich repeat (NBS-LRR) proteins including an N-terminal coiled-coil (CC) domain. The CC domain is completely conserved among these alleles, while sequence exchange is detected in the NBS and LRR regions, indicating intragenic recombination or gene conversion between alleles (Bourras et al., 2015). *Pm2* has been widely used in the world due to its significant resistance to powdery mildew in a long history (Jin et al., 2021). At *Pm2* locus, several alleles have been identified from a series of diversified genotypes (Jin et al., 2018). These alleles have significantly different resistance spectra to *Bgt* isolates, which is crucial to the study of genetic diversity and variation of *Pm2* and provides valuable resources for wheat disease resistance breeding. *Pm2a*, derived from American wheat cultivar Ulka, was cloned by mutant chromosome sequencing (MutChromSeq) for six ethylmethylsulfone (EMS)-induced mutants (Sánchez-Martín et al., 2016). *PmCH1357*, derived from wheat line CH1357, was map-based cloned combining with 90K SNP array. Further analysis showed that the sequence of *PmCH1357* was completely consistent with that of *Pm2a* (Chen et al., 2019).

Plants have evolved two layered immune systems to prevent invading pathogens (Dodds and Rathjen, 2010; Dangl et al., 2013). The first layer -, triggered by perception of microbe-associated molecular patterns (MAMPs) or pathogen-associated molecular patterns (PAMPs), is known as pattern-triggered immunity (PTI) (Jones and Dangl, 2006; Boller and Felix, 2009; Boller and He, 2009). The second layer is that intracellular nucleotide-binding leucine-rich-repeat (NLR) directly or indirectly perceives pathogen effectors and leads to effector-triggered immunity (ETI) (Cui et al., 2015; Zhang et al., 2017). ETI is governed by NLR and often accompanied by the occurrence of cell death around pathogen infection sites, which is believed to limit the spread of pathogens (Dangl et al., 2013; Jones et al., 2016). In wheat, nine of 13 cloned *Pm* genes, including *Pm1*, *Pm2a*, *Pm3*, *Pm5*, *Pm8*, *Pm17*, *Pm21*, *Pm41* and *Pm60*, encode NLR immune receptors that recognized pathogen effectors and activated ETI pathway. In general, NLRs are present in an inactive state without specific effectors (Praz et al., 2017). Intramolecular interactions could help to keep the NLR proteins in an autoinhibition state (Rairdan et al., 2008; Wang et al., 2015). However, the PTI and ETI pathways triggered by wheat powdery mildew resistance genes have been ambiguous.

NLR gene can regulate the disease resistance pathway by recruiting transcription factors (TFs) in plants. Transcription factors related to plant stress mainly include WRKY, MYB, NAC, bZIP and AP2/ERF. Plant WRKY TFs which are specific in higher plants play important roles in the regulation of transcriptional processes to modulate pathogen triggered cellular responses (Pandey and Somssich, 2009; Tsuda and Somssich, 2015). Some WRKY TFs, such as WRKY IIa subgroup members, have been shown to act as regulators of plant immunity. For instance, the chimeric protein AtWRKY52/RRS1, which possesses the dual structure and function of coiled-coil nucleotide-binding site leucine-rich repeat (CNL) protein and WRKY TF, interacts with the effector PopP2 in nucleus to be resistant to *Ralstonia solanacearum* in *Arabidopsis* (Tasset et al., 2022). Rice panicle blast gene *Pb1* encodes a CNL disease resistance protein which depends on interacting with WRKY45 in the nucleus to regulate the resistance (Inoue et al., 2013). *MLA10* interacts with *HvWRKY1/HvWRKY2* through the CC domain to relieve the inhibition of *HvWRKY1/HvWRKY2* on barley powdery mildew resistance, which makes barely plants resistant to powdery mildew (Maekawa et al., 2011). However, the interactions between *Pm* genes and transcription factors remain to be further elucidated in wheat.

Wheat breeding line KM2939 that carried *Pm2b* was highly resistant to powdery mildew at both seedling and adult stages. *Pm2b* was flanked by *Xcfd81* and *Xswgi011* with genetic distance 0.5 and 1.3 cM on the chromosome 5DS, respectively (Ma et al., 2015). In this study, we intend to map-based clone *Pm2b*, reveal the molecular mechanism of regulating resistance to powdery mildew by screening the interacted protein of PM2, then develop high-throughput and efficient markers to provide a reliable tool for marker-assisted selection breeding (MAS) of *Pm2b*.

## Materials and methods

### Plant materials and phenotype assessment of powdery mildew

The population of 4,354 F_2:3_ families, derived from a cross of the highly resistant wheat breeding line KM2939 and highly susceptible wheat cultivar Shimai 15 (SM15), was used to fine-map the powdery mildew resistance gene *Pm2b*. A collection of 133 wheat cultivars/lines was used to validate the specific marker of *Pm2b* in different genetic backgrounds (**Table S5**). Susceptible wheat cultivar Heng 4399 (H4399) was included in this study for maintaining and increasing *Bgt* isolates, and it was also used as the susceptible control. All plants were inoculated with the *Bgt* isolate E09 to assess the powdery mildew reaction in the greenhouse as previously described (Jin et al., 2021). When the pustules were fully developed on the first leaf of H4399 at about 14 days post inoculation (dpi), infection types (ITs) for each plant were assessed based on a 0-4 scale, and plants with ITs 0-2 were regarded as resistant and those with ITs 3 and 4 as susceptible (Si et al., 1987).

### Microscopic analyses of powdery mildew resistance reaction

Microscopic analyses were performed as previously described (Wang et al., 2014). The 2 cm leaf segments were cut at 14 dpi with inoculating *Bgt* isolate E09 and fixed at 37°C for 24 h in 2 ml of Carnoy’s Fluid (ethanol: acetic acid, 3:1, v/v), then stained with 2 mL of 0.6% (w/v) Coomassie blue solution for 3 min. Excess dye was rinsed off carefully with distilled water, then determined the percentage of microcolonies formed from the total number of germinated spores. Samples were observed under an Olympus BX-53 microscope (Olympus, Japan).

### DNA/RNA bulks construction and BSR-Seq analysis

The resistant and susceptible DNA bulks, which consisted of 30 homozygous resistant and 30 homozygous F_2:3_ families of the cross KM2939/SM15, were used in DNA-based bulked segregant Analysis (BSA) to validate polymorphic markers (Michelmore et al., 1991). Bulked segregant RNA-Sequencing (BSR-Seq) was conducted by Biomarker Technologies (Beijing, China). Thirty resistant and susceptible F_2:3_ families, represented by equal amounts of leaf tissues from each single plant of each family, were respectively selected randomly to compose the resistant bulk and susceptible bulk for BSR-Seq.

### DNA extraction and development of molecular markers

Total genomic DNA was extracted using cetyltrimethylammonium bromide (CTAB) method as previously described (Sharp et al., 1988). To convert candidate SNPs or Indels linked to *Pm2b* to available markers, 3 kb flanking sequences for each putatively linked SNPs or Indels were extracted from the IWGSC reference v2.1, used as reference sequence for developing marker. The primers were designed with software Primer 5.0.

### Construction of linkage map

Chi-squared (*χ^2^*) tests were performed to evaluate goodness of fit of observed phenotyping data from the expected segregation ratios of the F_2:3_ generations. The linkage relationship of polymorphic markers and *Pm2b* was established using Software MAPMAKER/Exp (version 3.0b) with the Kosambi map function and the logarithm of odd (LOD) score threshold was set at 3.0 (Kosambi, 1943). Putative genes were annotated with the TriAnnot pipeline and confirmed using the BLAST analysis tools available at NCBI and EnsemblPlants.

### Function verification by BSMV-VIGS

Barley stripe mosaic virus-induced gene silencing (BSMV-VIGS) was used to verify the gene function of *Pm2b* and *TaWRKY76-D*. The non-conserved-domain fragments of *Pm2b* and *TaWRKY76-D* were amplified with the specific primers (**Table S1**) from the cDNA of KM2939, digested with *EcoR*I/*Sal*I, and reversely inserted into BSMV: γ vector to generate the recombinant vectors BSMV: *Pm2b* and BSMV: *TaWRKY76-D*. Using the system established by He et al. (2018) with the following steps: at 7-12 days after virus inoculation, the plants were treated with *Bgt* isolate E09. About 14 dpi later, the third leaves with virus symptoms were collected for phenotypic identification, staining with Commassie blue for observation of fungal development and expression analysis with the quantitative real-time PCR (qRT-PCR) (**Table S1**). At least 10 leaves were analyzed in each experiment to evaluate the effect of gene silencing.

### Mutants analysis

About 5,000 seeds of KM2939 were treated with 0.8% Ethylmethylsulfone (EMS). Then, these seeds were sown in the field to generate M_1_ plants. To screen susceptible mutants, 30-50 seeds of each M_2_ family were evaluated for the response to *Bgt* isolate E09. The susceptible M_2_ plants were transplanted to the field to produce M_3_ generation for progeny testing to confirm the mutations. The genomic DNA sequence of candidate gene in each powdery mildew susceptible mutant was obtained by PCR with specific primers (**Table S1**), and then compared for nucleotide variations with wild type by multiple sequence alignment.

### RNA extraction and qRT-PCR analysis

Total RNA was extracted from wheat using TRIzol reagent (Invitrogen, USA). About 2 μg of RNA was used for reverse transcription with a FastQuant RT Kit (Tiangen, China). The qRT-PCR assays were performed using SYBR Premix Ex Taq (Takara, China) on the Bio-Rad CFX Connect real-time PCR system (BIO-RAD, USA). The relative expression of each gene was calculated as a fold change using the comparative CT method (Livak and Schmittgen, 2001). For each sample, three technical replications were analyzed. The *TaActin* was used as the internal control for normalization. Primers used in this study were listed in **Table S1**.

### Subcellular localization and Western blotting

The full-length coding sequence (CDS) of *Pm2b* was amplified from KM2939 seedling cDNA sample and sub-cloned into the pCAMBIA1305-GFP vector (Han et al., 2020). For PM2b protein subcellular localization, *Agrobacterium tumefaciens* strain GV3101 harboring proper recombinant vectors constructs were infiltrated into *N. benthamiana* leaves. The fluorescence signal of green fluorescent protein (GFP) was observed at 48 h after infiltration under a Leica TCS SP5 Confocal Microscope (Leica, Germany). The CDS of *TaWRKY76-D* was amplified and sub-cloned into the pJIT163-GFP vector (Liu et al., 2020). For TaWRKY76-D protein, the recombinant plasmid was each introduced into wheat protoplasts using the polyethylene glycol (PEG)-mediated method as previously described (Liu et al., 2020). After incubation for 16 h at 22°C, GFP fluorescence was observed under the confocal microscope.

Protein isolation and western blotting were performed as previously described (Gao et al., 2020). In brief, 0.1 g of *N. benthamiana* leaves were collected at 48 hpi, then homogenized in 300 μl of plant extraction buffer (CWBIO, China). Proteins were separated on 10% SDS-PAGE gels followed by western blotting analysis using anti-GFP antibody (Abclonal, China).

### Yeast two-hybrid assay and LCI assay

Yeast two-hybrid (Y2H) assays were performed as previously described (Wu and Li, 2017). The indicated fragments were amplified and sub-cloned into the PXGY17, PXGY18, pGBKT7 or pGADT7 vectors to generate the recombinant vectors. After the co-transformation of pGBKT7-fragment-A and pGADT7-fragment-B plasmids into yeast AH109 cells, or the co-transformation of PXGY17-fragment-A and PXGY18-fragment-B plasmids into yeast NMY51 cells, the interaction between indicated proteins was determined by the growth of the co-transformants on a selection medium (SD/-Trp/-Leu/-His/-Ade). For Y2H screening, full-length CDS of *Pm2b* was fused to pGBKT7 vector for bait construction and the screening of a wheat cDNA library derived from Yangmai 158 with inoculating *Bgt* isolate E09.

The Luciferase complementation imaging (LCI) assays for the interaction between indicated proteins in *N. benthamiana* leaves were performed as described previously (Liu et al., 2017). The indicated fragments were fused with the N-terminal or C-terminal regions of the LUC reporter gene, respectively, and transformed into *A. tumefaciens* strain GV3101. *Agrobacteria* harboring the nLUC and cLUC derivative constructs were co-infiltrated into *N. benthamiana*, and the LUC activity was imaged and analyzed at 48 hpi using the NEWTON7.0 Bio. Plant Imaging System (Vilber Bio Imaging, France).

## Results

### Map-based cloning of Pm2b

**Figure 1:**
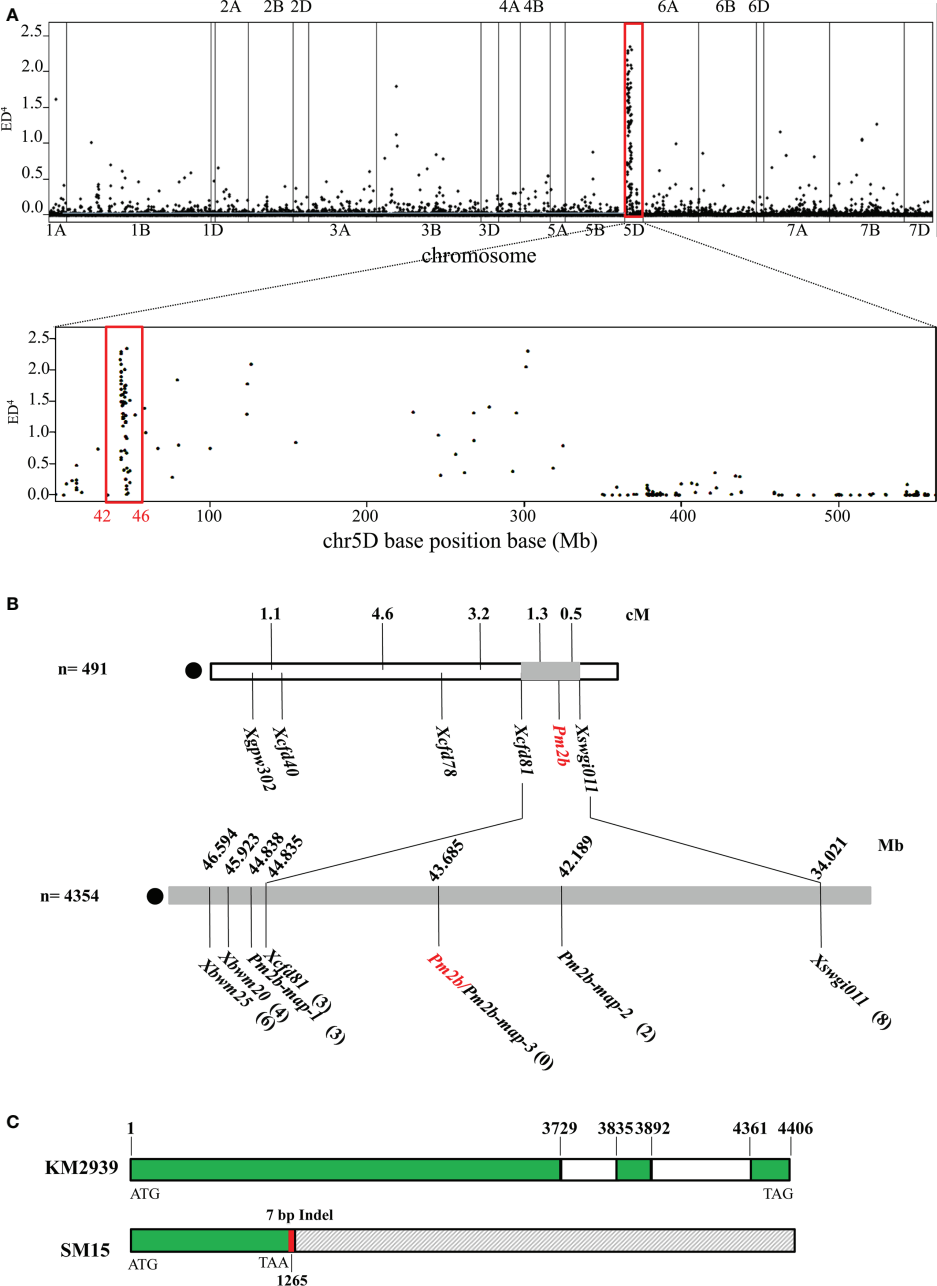
Map-based cloning of powdery mildew resistance gene *Pm2b*. **(A)** Distribution of polymorphic SNP loci between the resistant and susceptible bulks on 21 chromosomes and density of polymorphism SNP loci on chromosome 5D with 1 Mb intervals. **(B)** Linkage map of *Pm2b*. **(C)** Genomic structure of the candidate gene *TraesCS5D03G0111700* in KM2939 and Shimai 15 (SM15). The green box represents the exon, the white box represents the intron, the gray box represents the deletion in SM15, the red box represents the seven missing bases and the number represents the position.

Wheat breeding line KM2939 was resistant with an IT 0 to *Bgt* isolate E09, while wheat cultivar SM15 was susceptible with IT 4. The 4,354 F_2:3_ families segregated as 1,107 homozygous resistant, 2,208 segregating, and 1,029 homozygous susceptible, which fitted a 1:2:1 ratio (*χ^2^* = 3.99, *df* = 2, *P* = 0.14), indicating that *Pm2b* in KM2939 was a dominant powdery mildew resistance gene. To fine-map *Pm2b*, we first conducted the BSR-Seq analysis for KM2939, SM15, resistant and susceptible bulks. The 6.9 Gb, 9.0 Gb, 20.2 Gb and 23.1 Gb clean data were obtained from KM2939, SM15, resistant and susceptible bulks, respectively. The percentages of clean reads with a Q30 were 93.42%, 93.28%, 93.24% and 93.5%, respectively. The percentages of reads mapping to the reference genome were all more than 85%. These data indicated that the sequencing quality was high and suitable for subsequent analysis. A total of 1,198 candidate SNPs and InDels associated with the disease resistance were identified. The most abundant enrichment of the SNP loci was observed on chromosome 5D (**Figure 1A**). The density distribution of SNP loci showed a strong peak at the 42.41-46.86 Mb interval of the chromosome 5DS (**Figure 1A**), demonstrating a high probability of linkage with the resistance gene *Pm2b* in KM2939.

Based on sequence variations between resistant and susceptible bulks, three polymorphic markers *XPm2b-map-1*, *XPm2b-map-2*, and *XPm2b-map-3* in the region were developed and showed consistent polymorphism in resistant and susceptible parents and DNA bulks. Combining with previously reported markers *Xcfd81*, *Xswgi011*, *Xbwm2*0 and *Xbwm25*, these seven markers were used to validate in the 4,354 F_2:3_ families. Finally, *Pm2b* was flanked by *Xcfd81* and *Pm2b-map-2* with a physical region of 1.64 Mb, and marker *Pm2b-map-3* was co-segregated with *Pm2b* (**Figure 1B**, **Table S1**). According to the fine-mapped interval and gene annotation of IWGSC_v2.1_HC_gene, there were 20 high confidence genes. However, only *TraesCS5D03G0111700* existed variations in CDS between resistant parent KM2939 and susceptible parent SM15 (**Figure 1C**, **Table S2**). More importantly, *TraesCS5D03G0111700* in SM15 contained a 7 bp deletion in the first exon, which formed a new termination site at the 422^th^ triplet of the shifted reading frame (**Figure 1C**, **Table S2**). Therefore, *TraesCS5D03G0111700* was the important candidate of *Pm2b*. And the sequence of *TraesCS5D03G0111700* in resistant parent KM2939 was identical to *Pm2a* and *PmCH1357* cloned previously.

### Functional verification of Pm2b

**Figure 2:**
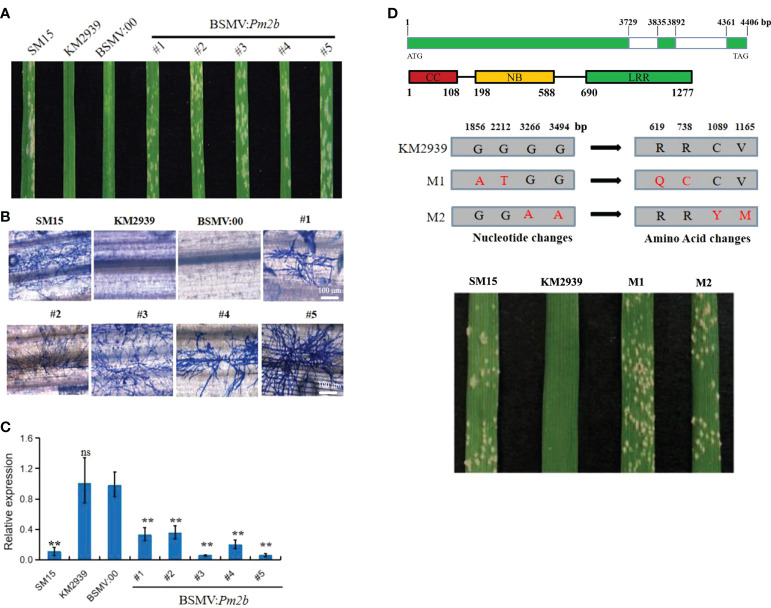
Function verification of powdery mildew resistance gene *Pm2b*. Macroscopic phenotypes **(A)**, microscopic phenotypes **(B)** and expression analysis of *Pm2b* **(C)** after silencing by barley stripe mosaic virus-induced gene silencing (BSMV-VIGS) of *Pm2b* in wheat line KM2939. Bar = 100 μm. Error bars represent SD of three independent experiments. Statistically significant differences (Student’s *t*-test): ns, not significant; **, *P <*0.01. **(D)** The analysis on two independent susceptible mutants of KM2939. The upper Figure indicated gene and protein structures of *Pm2b*, the middle Figure indicated the nucleotide and amino acid changes of *Pm2b* in two susceptible mutants M1 and M2, the below Figure indicated response of M1 and M2 to *Blumeria graminis* f. sp. *tritici* (*Bgt*) isolate E09.

To identify that *TraesCS5D03G0111700* is essential to *Pm2b* resistance to powdery mildew, the silence effect of *TraesCS5D03G0111700* in KM2939 was analyzed by BSMV-VIGS. The non-conserved-domain sequence, amplified by the primer Pm2b-VIGS, was designed as silencing target (**Table S1**). The qRT-PCR analysis showed that the expression level of *TraesCS5D03G0111700* had a 66-95% decreasing in plants treated with BSMV: *Pm2b*, revealing a high efficiency of BSMV-VIGS system (**Figure 2C**). Fifteen of twenty plants which were treated with BSMV: Pm2b showed susceptible to *Bgt* isolate E09 with IT 4. However, no spore could be observed on leaves of empty vector BSMV: 00 (**Figures 2A, B**). The results indicated that silencing of *TraesCS5D03G0111700* could compromise the resistance of KM2939.

To further verify the function of *TraesCS5D03G0111700*, we obtained two EMS independent susceptible mutants of KM2939 (**Figure 2D**). *TraesCS5D03G0111700* was cloned from the two mutants to compare their differences with that of KM2939. Two SNPs were identified in the LRR domain of mutant M1, which caused Arg619Gln and Arg738Cys substitution. Two SNPs were identified in the LRR domain of mutant M2, which caused Cys1089Tyr and Val1165Met substitution (**Figure 2D**). In conclusion, *TraesCS5D03G0111700* was the *Pm2b* using BSMV-VIGS and mutant analysis in KM2939.

### Expression patterns of Pm2b

**Figure 3:**
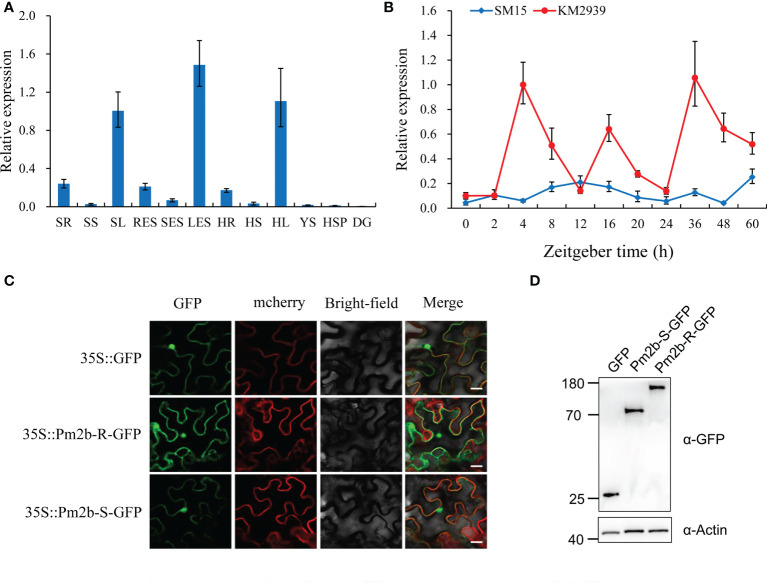
Expression patterns of powdery mildew resistance gene *Pm2b*. **(A)** Tissue specific expression patterns of *Pm2b* in various KM2939 tissues. Seedling roots (SR), seedling stems (SS), seedling leaves (SL), roots at the elongation stage (RES), stems at the elongation stage (SES), leaves at the elongation stage (LES), roots at the heading stage (HR), stems at the heading stage (HS), leaves at the heading stage (HL), young spikes (YS), spikes at the heading stage (HSP) and grains (DG). **(B)** Expression pattern of *Pm2b* after inoculating with *Blumeria graminis* f. sp. *tritici* (*Bgt*) isolate E09 at 2, 4, 8, 12, 16, 20, 24, 26, 48 and 60 hours post inoculation (hpi). In B and C, normalized values of *Pm2b* expression relative to *Actin* were given as mean ± SD from three replicates. **(C)** Subcellular localization of *Pm2b* in *Nicotiana benthamiana*. Transient expression of GFP and *Pm2b* driven by CaMV 35S promoter in *N. benthamiana*. Bar = 10 μm. **(D)** Protein expression levels of Pm2b-R and Pm2b-S shown by western blotting. Total proteins were extracted from *N. benthamiana* leaves at 48 hpi and the tagged proteins were detected by western blotting using an anti-GFP antibody (α-GFP). Equal protein loading is shown by immunoblot detection of actin (α-Actin) throughout this article.

The qRT-PCR was performed to identify the expression pattern of *Pm2b* in different tissues of KM2939 (**Table S1**). The transcript level of *Pm2b* was the highest in leaves, slightly lower in roots and substantially lower in stems, spikes and developing grains (**Figure 3A**). Next, we further used qRT-PCR to investigate the expression pattern of *Pm2b* in the KM2939 and SM15 leaves after inoculating with the *Bgt* isolate E09 at different time. As shown in **Figure 3B**, the transcript level of *Pm2b* was rapidly up-regulated and reached a peak at 4 h after inoculation which was about 10-fold compared with non-inoculated KM2939 at 0 h. However, the expression of *Pm2b* was declined and reversed to 0.51- to 0.14- fold from 8 to 12 h, compared with the inoculated KM2939 at 4 h. In contrast, the transcript level of *Pm2b* in SM15 did not change significantly after inoculation during 0-60 h, compared with KM2939 (**Figure 3B**).

### Subcellular localization of Pm2b

To identify the subcellular localization of PM2b, the CDS of *Pm2b* was fused with vector pCAMBIA1305-GFP and the recombinant plasmid was infiltrated into *N. benthamiana* leaves using *Agrobacterium*-mediated transient expression. As shown in **Figure 3C**, the GFP fluorescence patterns of the fused pCAMBIA1305-Pm2b-GFP proteins were distributed throughout the nucleus, cytoplasm and plasma membrane. Western blotting showed that Pm2b-S-GFP and Pm2b-R-GFP fusion proteins were properly expressed in *N. benthamiana* leaves (**Figure 3D**).

### Domains interaction of PM2b

**Figure 4:**
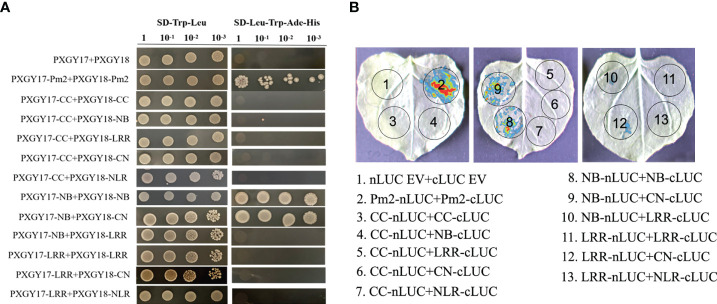
Interactions of different domains of PM2b. **(A)** The protein interaction of different domains of PM2b by yeast two-hybrid. Different domains of PM2b were fused to DNA binding domain (BD) and activation domain (AD) of GAL4, respectively. The yeast strains AH109 were serially diluted (10^0^-10^-3^) before spotting on selection medium. SD/-Trp/-Leu, synthetic dextrose medium lacking Trp and Leu; SD/-Trp/-Leu/-His/-Ade, synthetic dextrose medium lacking Trp, Leu, His and Ade. **(B)** The protein interaction of different domains of PM2b by luciferase complementation imaging (LCI) assays. The N- or C-terminal fragment of LUC (nLUC or cLUC) was fused with the indicated proteins. The indicated constructs were co-expressed in *Nicotiana benthamiana* by agroinfiltration. Images of chemiluminescence were recorded by applying 0.5 mM luciferin 48 h after infiltration. Similar results were obtained in three biological replicates.

To identify whether the PM2b was self-associated, we conducted Y2H and LCI assays. Firstly, the CC, NB, LRR, CN, NL and PM2b domains were constructed into yeast vectors PXGY17 and PXGY18, respectively, to co-transformation yeast cells. These results suggested that PM2b could self-associate, NB domain self-associated and interacted with CN (**Figure 4A**). Then, using the LCI assays in *N. benthamiana* cells, it was found that the co-infiltration of Pm2b-nLUC and cLUC-Pm2b, NB-nLUC and cLUC-NB, Pm2b-NB and cLUC-CN resulted in luminescence generated by the complemented luciferase (**Figure 4B**). In conclusion, PM2b could self-associate through NB domain.

### PM2b interacts with TaWRKY76-D

**Figure 5:**
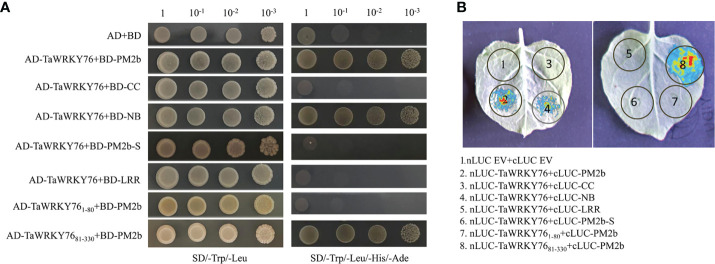
Protein-protein interactions between PM2b and TaWRKY76-D. **(A)** Yeast two-hybrid assay showed the interaction between PM2b and TaWRKY76-D. BD: GAL4 DNA binding domain; AD: GAL4 activation domain. The yeast strains AH109 were serially diluted (10^0^-10^-3^) before spotting on selection medium. SD/-Trp/-Leu, synthetic dextrose medium lacking Trp and Leu; SD/-Trp/-Leu/-His/-Ade, synthetic dextrose medium lacking Trp, Leu, His and Ade. **(B)** Luciferase complementation imaging (LCI) assays showed the interaction between PM2b and TaWRKY76-D in *Nicotiana benthamiana*. nLUC, N-terminal part of LUC; cLUC, C-terminal part of LUC. The nLUC and cLUC derivative constructs were coexpressed in *N. benthamiana* by agroinfiltration. Images of chemiluminescence were recorded by applying 0.5 mM luciferin 48 h after infiltration. Similar results were obtained in three biological replicates.

To identify additional components of Pm2b-mediated immune reaction, a cDNA library of *Bgt*-infected leaves was screened with the bait of PM2b. TaWRKY76-D was identified as a candidate interactor by PM2b bait (**Table S3**). Targeted Y2H analysis further confirmed that PM2b interacted with TaWRKY76-D, NB domain of PM2b interacted with TaWRKY76-D and PM2b interacted with WRKY domain of TaWRKY76-D, whereas PM2-SM15 did not interact with TaWRKY76-D (**Figure 5A**). We further performed a LCI assay in *N. benthamiana* cells to verify the interaction between PM2b and TaWRKY76-D. The co-infiltration of Pm2b-nLUC and cLUC-TaWRKY76-D, Pm2b-NB-nLUC and cLUC-TaWRKY76-D, Pm2b-nLUC and cLUC-WRKY-TaWRKY76-D resulted in luminescence generated by the complemented luciferase, which provided further evidence for interaction of PM2b and TaWRKY76-D *in vivo* (**Figure 5B**).

### Subcellular localization of TaWRKY76-D

**Figure 6:**
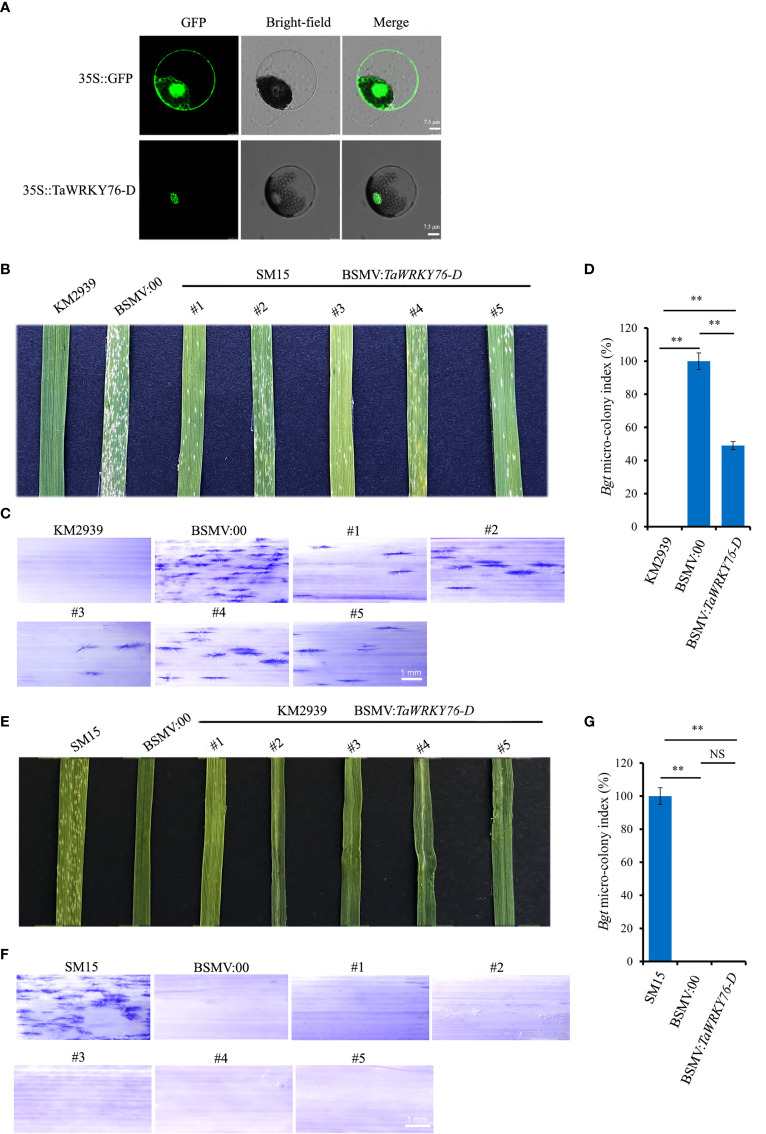
Subcellular localization and barley stripe mosaic virus-induced gene silencing (BSMV-VIGS) of *TaWRKY76-D*. **(A)** Subcellular localization of TaWRKY76-D protein in wheat protoplast. Transient expression of GFP and TaWRKY76-D driven by CaMV 35S promoter in wheat protoplast. Bar = 7.5 μm. **(B–G)** *TaWRKY76-D* negatively regulates wheat resistance to powdery mildew (*Blumeria graminis* f. sp. *tritici*, *Bgt*). Inoculated with *Bgt* isolate E09 at 14 days post inoculation, macroscopic **(B)**, microscopic **(C)** phenotypes and micro-colony index **(D)** after silencing by barley stripe mosaic virus-induced gene silencing (BSMV-VIGS) of *TaWRKY76-D* in wheat cultivar Shimai 15 (SM15), macroscopic **(E)**, microscopic phenotypes **(F)** and micro-colony index **(G)** after silencing by BSMV-VIGS of *TaWRKY76-D* in wheat line KM2939. Bar = 1 mm, Error bars represent SD of three independent experiments. Statistically significant differences (Student’s *t*-test): ns, not significant; **, *P <*0.01.

To investigate the subcellular localization of TaWRKY76-D in wheat protoplasts, the CDS of the *TaWRKY76-D* was fused in-frame with the gene encoding GFP, and the resulting constructs were introduced into wheat protoplasts. As shown in **Figure 6A**, the GFP fluorescence pattern of the fused TaWRKY76-D protein was distributed on nucleus.

### Functional verification of TaWRKY76-D

To identify the function of *TaWRKY76-D* in wheat immunity against powdery mildew, we conducted BSMV-VIGS to silence *TaWRKY76-D* in resistant parent KM2939 and susceptible parent SM15, respectively. The non-conserved-domain sequence of *TaWRKY76-D* was designed as silencing target (**Table S1**). The amounts of spores on the leaf of SM15 treated with BSMV: *TaWRKY76-D* reduced almost one half compared with that treated with BSMV: 00 (**Figures 6B–D**). No spore was observed on the leaves of KM2939 treated with BSMV: *TaWRKY76-D* and BSMV: 00 (**Figures 6E–G**). The *TaWRKY76-D* transcript levels were reduced by 66-86% and 78-85% in SM15 and KM2939 treated with BSMV: *TaWRKY76-D*, respectively (**Figure S1**). Thus, silencing of *TaWRKY76-D* increased powdery mildew resistance to the *Bgt* isolate E09.

### Development and verification of specific marker of Pm2b

To determine the variations of *Pm2b* locus, we cloned and sequenced the entire genomic region of *Pm2* from 15 wheat cultivars/lines which were reported to carry *Pm2* alleles, and seven susceptible wheat cultivars without any allele of *Pm2* (**Table S4**). The results showed that the sequences of 15 documented *Pm2* alleles were identical to *Pm2b*, whereas the sequences of seven susceptible cultivars were the same as Pm2-SM15 (**Table S4**). There were several non-synonymous SNPs between KM2939 and SM15 in the first exon of NLR (**Table S2**). According to these differential SNPs, a high-throughput and high-efficiency KASP (Kompetitive Allele Specific PCR) marker *K529* based on the variation of c.556C>A was developed to detect the presence of *Pm2b*. Subsequently, 133 wheat cultivars/lines from the main wheat producing regions were tested with marker *K529*. *K529* could consistently amplify specific genotypes differed from KM2939 in 105 out of 133 wheat accessions (**Figure S2**, **Table S5**). Therefore, comparing with the common PCR markers established previously, KASP marker *K529* could be used in MAS with high throughput and efficiency for detecting *Pm2b* in those wheat genetic backgrounds.

## Discussion

### Three wheat powdery mildew resistance genes are encoded by a unique sequence

Recent studies showed that 393 of 659 powdery mildew-resistant accessions carried *Pm2*, including 83 cultivars, 252 advanced breeding lines, three landraces and 33 introduced cultivars (Jin et al., 2021). Cloning and revealing the disease resistance mechanism of *Pm2* could provide theoretical basis for its application in production. *Pm2a* and *PmCH1357* were cloned by different means and their sequences were identical (Sánchez-Martín et al., 2016; Chen et al., 2019). But the further studies of *Pm2* will be needed to verify its function and determine the disease resistance mechanism. In the current study, we cloned *Pm2b* from wheat breeding line KM2939 using map-based cloning combined with BSR-Seq analysis. Subsequently, the function of *Pm2b* was verified by BSMV-VIGS assay and mutant analysis in KM2939. Sequence alignment showed that the genomic sequence of *Pm2b* was completely identical with *Pm2a/PmCH1357*. In conclusion, the cloning and function verification of *Pm2* further facilitated its application in wheat powdery mildew resistance breeding. In addition, in this study, the marker *K529* was developed based on the SNP on the first exon of *Pm2b* between resistant and susceptible parents and was verified in 133 wheat cultivars/lines. The marker *K529* was able to identify *Pm2b* in a high throughput and efficiency scale during the process of MAS.

### Significance of homologous polymers of Pm2b

Several studies have shown that *R* gene-mediated disease resistance against pathogens depended on the formation of heterologous or homologous polymers in plants (Jones et al., 2016). For instance, two different CC-NBS-LRR genes form a paired NB-LRR receptor, which is required for resistance to leaf rust resistance in tetraploid and hexaploid wheat (Loutre et al., 2009). In a recent example, the NB-LRR gene *PigmR* confers broad-spectrum resistance to rice blast in *Magnaporthe oryzae*, whereas the paired NB-LRR *PigmS* suppresses *PigmR*-mediated resistance by competitively affecting *PigmR* homodimerization to regulate resistance (Deng et al., 2017). In barley, MLA receptors self-associate *in vivo*, but self-association appears to be independent of effector-triggered receptor activation, further study showed dimeric CC module in downstream immune signaling (Maekawa et al., 2011). In the current study, we found that PM2b could form homologous dimer through NB and NB domain interaction. The results showed that *Pm2* induced a strong hypersensitive cell death response upon recognition of *AvrPm2* whereas no hypersensitive reaction was observed when *Pm2* was co-expressed with the GUS control in *N. benthamiana* (Praz et al., 2017). It was speculated that *Pm2b* existed in the form of homologous polymers and maintained autoinhibition in the absence of pathogen effector, and *Pm2b* function might depend on directly or indirectly recognizing the specific effector *AvrPm2* to activate the immunity.

### PM2b interacting with TaWRKY76-D to regulate powdery mildew resistance

Plant WRKY transcription factors play important roles in the regulation of transcriptional processes to modulate pathogen-triggered cellular responses (Tsuda and Somssich, 2015; Han et al., 2020). For instance, barley HvWRKY1 and HvWRKY2 were shown to interact with MLA which encoded a NLR receptor in the nucleus to derepress barley immunity and potentiate ETI responses (Shen et al., 2007). In *Arabidopsis*, the RPS4 and RRS1 proteins formed a paired NB-LRR receptor, which was required for resistance to bacterial and fungal pathogens. In this immune receptor complex, the WRKY domain of RRS1 served as a decoy to bind the pathogen effector and initiated resistance signaling mediated by RPS4 (Le Roux et al., 2015; Sarris et al., 2015). In our study, we identified a WRKY transcription factor TaWRKY76-D by Y2H, and PM2b could physically interact with TaWRKY76-D through NB domain and WRKY domain interaction. Gene silencing of *TaWRKY76-D* showed that it negatively regulated powdery mildew resistance in wheat. It was likely that *Pm2b* interacted with TaWRKY76-D to remove the inhibition of TaWRKY76-D on downstream genes. Previous study reported that PM2 could interact with its avirulent gene AVRPM2 directly or indirectly to induce strong hypersensitive reaction in *N. benthamiana*, when infected with *Bgt* isolates (Praz et al., 2017). We speculated that AVRPM2 enhanced the interaction between PM2 and TaWRKY76-D to strengthen the resistance to powdery mildew. Similarly, WRKY76-D could facilitate the recognition of PM2 and AVRPM2 through interacting with PM2 to initiate the disease resistance response. However, silencing *TaWRKY76-D* did not fully gain absolute resistance to powdery mildew. Therefore, *TaWRKY76-D* could be the important component in PM2b-mediated powdery mildew resistance pathway.

## Conclusion

In conclusion, we cloned powdery mildew resistance gene *Pm2b* from resistant line KM2939. The function of *Pm2b* was verified by barley stripe mosaic virus-induced gene silencing technology and loss-of-function mutants. *Pm2b* encoded the coiled-coil nucleotide-binding site leucine-rich repeat protein. PM2b could self-associate through the NB domain. Notably, we identified PM2b interacting with the transcription factor TaWRKY76-D, which depended on the NB domain of PM2b and WRKY domain of TaWRKY76-D. TaWRKY76-D negatively regulated the resistance to powdery mildew. In addition, we developed a KASP marker *K529* which could take the advantage of high-throughput and high-efficiency for detecting *Pm2b* and be useful in molecular marker assisted-selection breeding. This study could provide theoretical basis for wheat disease resistance breeding.

## Data availability statement

The original contributions presented in the study are included in the article/**Supplementary Material**. Further inquiries can be directed to the corresponding authors.

## Author contributions

DA and LL conceived the project. YJ, HL, and DA designed the experiments. YJ, HL, TG, LX, GH, PM, XL, YZ, and JF performed experiments. YJ and HL analyzed data and wrote the manuscript. DA and LL edited the manuscript and supervised the project. All authors contributed to the article and approved the submitted version.

## Funding

This research was supported by the National Key Research and Development Program of China (2021YFD1200600), the National Natural Science Foundation of China (32272105), and the Hebei Province Key Research and Development Program (22326306D).

## Acknowledgments

The authors are grateful to Hailiang Mao from Huazhong Agricultural University of China for providing cDNA library of Yangmai 158.

## Conflict of interest

The authors declare that the research was conducted in the absence of any commercial or financial relationships that could be construed as a potential conflict of interest.

The reviewer PY declared a shared affiliation with the authors XL, YZ, JF, LL to the handling editor at the time of the review.

## Publisher’s note

All claims expressed in this article are solely those of the authors and do not necessarily represent those of their affiliated organizations, or those of the publisher, the editors and the reviewers. Any product that may be evaluated in this article, or claim that may be made by its manufacturer, is not guaranteed or endorsed by the publisher.

## Supplementary material

Supplementary Figure 1 Relative expression of *TaWRKY76* after silencing by barley stripe mosaic virus-induced gene silencing of *TaWRKY76* in wheat cultivar Shimai 15 **(A)** and line KM2939 **(B)**. Error bars represent SD of three independent experiments. Statistically significant differences (Student’s t-test): ns, not significant; **, *P <*0.01.

Supplementary Figure 2 Partial genotyping results of marker *K529* by Kompetitive Allele Specific PCR (KASP) assay in 133 wheat cultivar/lines. Orange circles represent the accessions that carry *Pm2b* (allele1/allele1), blue squares represent the accessions that did not carry *Pm2b* (allele2/allele2), and black diamonds represent the nontemplate control.
