# Supplementary figures and images for: PM2b, a CC-NBS-LRR protein, interacts with TaWRKY76-D to regulate powdery mildew resistance in common wheat
Source: Front Plant Sci. 2022 Oct 26;13:973065. doi: 10.3389/fpls.2022.973065 (PMC9644048; doi:10.3389/fpls.2022.973065)

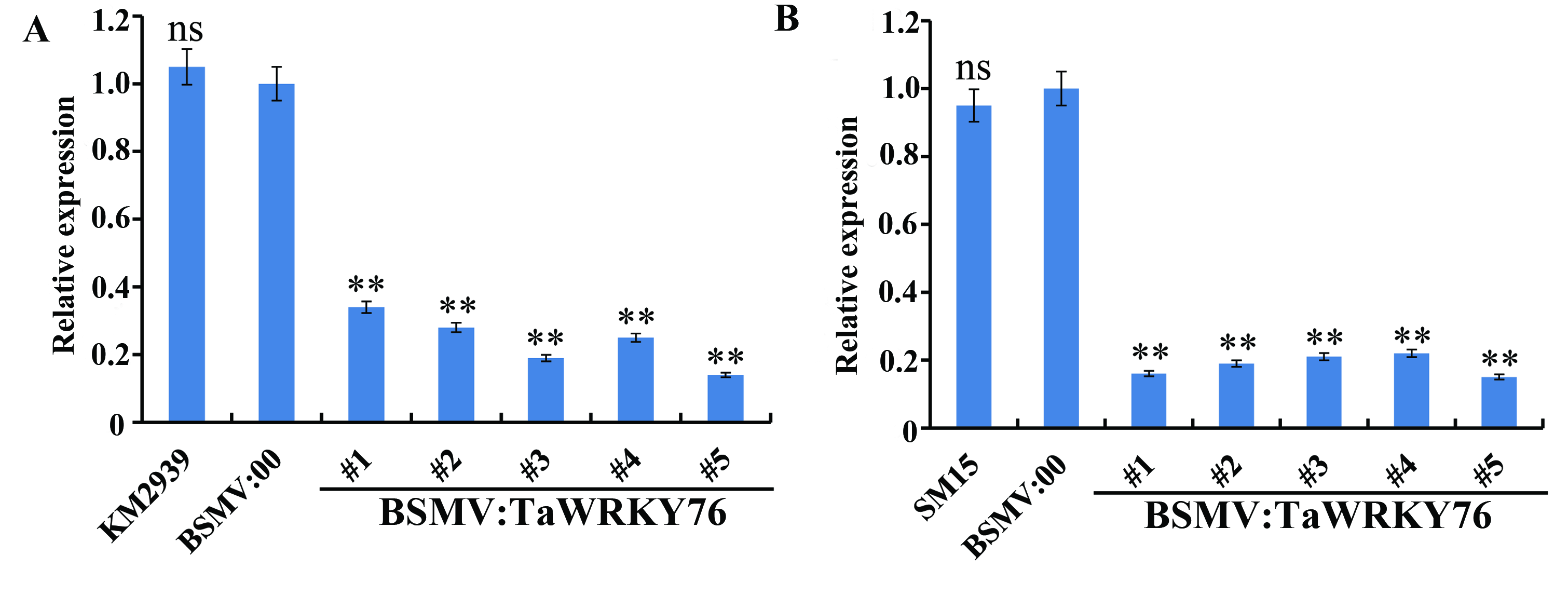

Supplement: Supplementary Figure 1 — Relative expression of TaWRKY76 after silencing by barley stripe mosaic virus-induced gene silencing of TaWRKY76 in wheat cultivar Shimai 15 (A) and line KM2939 (B). Error bars represent SD of three independent experiments. Statistically significant differences (Student’s t-test): ns, not significant; **, P <0.01. [file Image_1.tif]

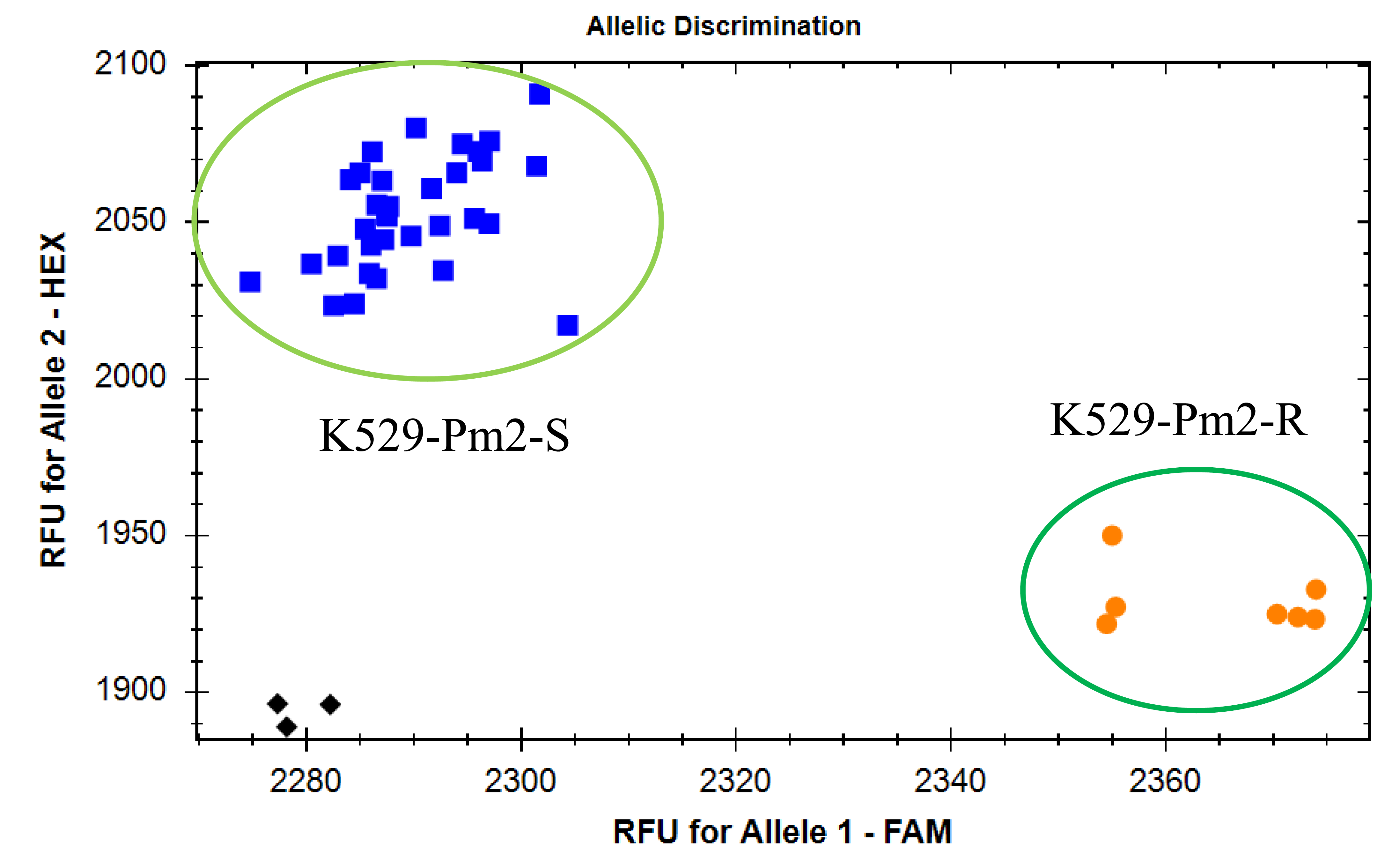

Supplement: Supplementary Figure 2 — Partial genotyping results of marker K529 by Kompetitive Allele Specific PCR (KASP) assay in 133 wheat cultivar/lines. Orange circles represent the accessions that carry Pm2b (allele1/allele1), blue squares represent the accessions that did not carry Pm2b (allele2/allele2), and black diamonds represent the nontemplate control. [file Image_2.tif]
